# Supplementary material for: Malaria Temporal Variation and Modelling Using Time-Series in Sussundenga District, Mozambique
Source: Int J Environ Res Public Health. 2021 May 26;18(11):5692. doi: 10.3390/ijerph18115692 (PMC8198511; doi:10.3390/ijerph18115692)
Supplement: Supplementary file 1 [file ijerph-18-05692-s001.zip › ijerph-1162463-supplementary.pdf]

	Year	Weeks	Sussundeng	Munhinga	Dombe	C. Mavuzi	Bunga	Darue
	0 a 4	0 a 4	0 a 4	0 a 4	0 a 4	0 a 4	0 a 4	0 a 4
2015	1	w1	387	83	31	86	21	29
	2	w2	341	283	45	96	29	100
	3	w3	558	48	110	137	23	89
	4	w4	504	68	135	100	25	47
	5	w5	411	67	119	100	31	64
	6	w6	329	21	95	56	26	83
	7	w7	293	160	61	63	38	68
	8	w8	314	67	93	61	20	45
	9	w9	362	142	144	71	31	81
	10	w10	339	126	85	71	23	46
	11	w11	404	92	111	83	23	29
	12	w12	252	140	145	98	31	38
	13	w13	325	45	74	89	23	61
	14	w14	302	87	53	75	29	47
	15	w15	271	71	92	60	31	59
	16	w16	275	55	93	89	26	81
	17	w17	292	85	20	81	24	53
	18	w16	273	111	54	108	28	54
	19	w19	283	133	82	97	30	61
	20	w20	251	98	85	66	28	77
	21	w21	177	103	60	121	28	73
	22	w22	241	84	91	104	30	61
	23	w23	217	110	150	49	28	58
	24	w24	204	92	103	59	28	66
	25	w25	177	69	40	63	29	64
	26	w26	179	52	34	56	29	64
	27	w27	200	67	26	63	24	45
	28	w28	146	75	38	52	24	45
	29	w29	131	53	42	46	29	31
	30	w30	133	62	43	38	31	37
	31	w31	128	49	49	35	21	32
	32	w32	131	38	23	32	27	24
	33	w33	103	62	61	42	29	37
	34	w34	100	53	43	34	22	32
	35	w35	109	26	34	30	22	34
	36	w36	65	29	42	25	29	52
	37	w37	112	36	55	35	27	42
	38	w38	105	48	47	35	30	23
	39	w39	50	26	51	61	30	25
	40	w40	110	67	32	47	29	41
	41	w41	145	52	92	51	24	37
	42	w42	191	83	91	59	32	32
	43	w43	192	97	68	64	26	47
	44	w44	183	107	58	53	27	30
	45	w45	165	87	81	42	25	49
	46	w46	232	77	78	42	27	37

	47	w47	207	41	61	48	27	52
	48	w48	178	49	63	53	31	30
	49	w49	179	85	37	51	27	30
	50	w50	326	119	30	68	30	44
	51	w51	263	110	57	68	28	13
2016	52	w52	303	159	44	68	27	13
	1	w1	369	148	84	101	31	32
	2	w2	381	143	49	86	27	45
	3	w3	252	87	51	61	31	38
	4	w4	312	88	31	57	24	35
	5	w5	390	136	15	56	22	50
	6	w6	380	184	0	60	21	23
	7	w7	443	167	73	96	13	18
	8	w8	367	158	79	73	18	70
	9	w9	302	89	45	52	18	42
	10	w10	282	78	82	96	32	54
	11	w11	247	120	52	65	29	52
	12	w12	251	113	46	67	31	28
	13	w13	306	229	76	93	22	34
	14	w14	249	76	93	56	13	62
	15	w15	232	114	81	99	29	85
	16	w16	321	152	106	99	19	44
	17	w17	290	31	68	82	31	31
	18	w16	283	74	56	51	22	41
	19	w19	226	115	76	74	17	40
	20	w20	275	101	87	73	34	28
	21	w21	203	141	80	68	20	3
	22	w22	295	20	87	48	36	21
	23	w23	212	0	92	56	24	55
	24	w24	209	60	57	73	29	40
	25	w25	170	68	45	65	28	38
	26	w26	185	77	61	71	25	27
	27	w27	98	46	78	72	29	28
	28	w28	167	94	54	61	27	31
	29	w29	162	41	87	65	33	29
	30	w30	164	51	89	68	31	28
	31	w31	128	46	56	69	27	37
	32	w32	150	16	78	71	23	19
	33	w33	160	19	83	78	24	29
	34	w34	141	33	107	61	33	25
	35	w35	118	30	85	53	0	30
	36	w36	83	53	56	74	32	22
	37	w37	104	35	64	59	26	30
	38	w38	124	38	104	71	26	20
	39	w39	151	16	71	82	0	28
	40	w40	193	31	46	60	33	25
	41	w41	152	24	14	74	21	23
	42	w42	193	41	28	68	28	25
	43	w43	207	72	34	62	13	30
	44	w44	208	86	22	66	13	21

	45	w45	261	89	30	71	15	26
	46	w46	272	75	28	60	16	29
	47	w47	238	69	36	63	16	24
	48	w48	215	59	20	58	20	33
	49	w49	252	81	36	68	20	31
	50	w50	135	80	32	73	14	35
	51	w51	179	73	32	66	15	34
	52	w52	163	76	41	59	15	23
2017	1	w1	451	128	136	90	33	110
	2	w2	483	139	102	64	9	113
	3	w3	472	113	104	21	36	80
	4	w4	385	167	101	37	19	98
	5	w5	303	188	72	43	27	70
	6	w6	330	180	69	51	21	65
	7	w7	207	125	128	51	34	70
	8	w8	326	88	115	27	31	94
	9	w9	292	36	93	47	21	52
	10	w10	239	110	103	49	25	74
	11	w11	200	51	135	58	23	72
	12	w12	263	100	116	52	26	77
	13	w13	268	62	118	63	26	92
	14	w14	248	17	91	27	20	76
	15	w15	212	41	128	36	31	50
	16	w16	183	54	46	69	33	86
	17	w17	211	2	82	20	36	47
	18	w16	201	117	90	80	29	63
	19	w19	223	47	100	53	32	110
	20	w20	196	101	141	29	28	79
	21	w21	179	65	152	42	24	66
	22	w22	179	56	152	42	27	59
	23	w23	99	56	139	16	31	58
	24	w24	73	6	78	26	32	45
	25	w25	74	30	80	41	36	61
	26	w26	65	27	112	54	29	43
	27	w27	103	31	70	45	32	21
	28	w28	49	78	140	32	40	57
	29	w29	58	42	56	21	16	31
	30	w30	58	21	99	47	32	29
	31	w31	297	74	122	21	18	53
	32	w32	33	15	122	26	30	38
	33	w33	68	16	102	35	31	52
	34	w34	24	33	93	16	6	53
	35	w35	47	30	109	36	23	44
	36	w36	48	20	88	12	20	59
	37	w37	62	20	117	29	22	50
	38	w38	88	15	117	26	19	42
	39	w39	60	10	105	22	20	31
	40	w40	46	22	92	25	21	28
	41	w41	41	21	104	39	21	29
	42	w42	36	28	58	28	22	47

	43	w43	32	30	98	17	28	34
	44	w44	53	5	47	41	19	53
	45	w45	117	13	21	39	19	10
	46	w46	88	40	44	11	20	15
	47	w47	78	54	46	33	18	23
	48	w48	92	53	49	21	25	39
	49	w49	211	58	86	26	50	65
	50	w50	216	89	96	31	53	94
	51	w51	268	30	86	23	17	60
	52	w52	351	60	96	29	49	49
2018	1	w1	320	12	86	51	42	63
	2	w2	362	62	102	69	79	54
	3	w3	321	97	102	31	95	77
	4	w4	281	58	52	32	32	79
	5	w5	207	89	67	36	79	59
	6	w6	213	54	90	0	91	73
	7	w7	187	54	90	35	59	49
	8	w8	142	57	130	39	0	63
	9	w9	210	67	60	14	86	48
	10	w10	301	36	131	75	0	63
	11	w11	127	72	75	23	64	69
	12	w12	178	78	123	13	21	64
	13	w13	161	68	106	26	64	41
	14	w14	62	113	79	39	73	0
	15	w15	133	122	72	36	84	44
	16	w16	236	118	92	76	70	69
	17	w17	200	91	122	57	58	66
	18	w16	187	104	94	57	72	48
	19	w19	175	91	106	65	82	45
	20	w20	157	61	98	29	87	32
	21	w21	172	64	141	51	77	36
	22	w22	157	42	99	17	72	36
	23	w23	90	64	88	43	0	43
	24	w24	72	73	90	42	67	64
	25	w25	49	46	78	36	72	45
	26	w26	30	40	59	17	76	44
	27	w27	37	46	59	26	64	32
	28	w28	63	42	51	18	64	57
	29	w29	48	33	76	29	43	61
	30	w30	62	0	55	15	38	30
	31	w31	33	41	0	0	0	0
	32	w32	0	0	0	0	0	30
	33	w33	41	0	0	16	0	51
	34	w34	29	24	81	0	55	0
	35	w35	36	27	0	0	0	0
	36	w36	125	36	110	22	42	39
	37	w37	80	69	104	38	51	45
	38	w38	77	67	79	30	47	54
	39	w39	55	39	90	16	40	30
	40	w40	102	42	88	30	34	22

	41	w41	70	53	66	10	47	31
	42	w42	112	32	71	31	40	36
	43	w43	57	0	60	24	51	37
	44	w44	149	63	31	24	39	16
	45	w45	87	81	92	27	33	51
	46	w46	151	94	67	34	52	66
	47	w47	117	34	88	41	50	36
	48	w48	188	148	88	31	50	28
	49	w49	271	37	73	56	60	19
	50	w50	263	153	62	43	49	32
	51	w51	82	110	49	9	42	18
	52	w52	226	110	15	27	35	41
2019	1	w1	297	193	59	11	21	42
	2	w2	371	212	67	10	29	88
	3	w3	392	293	73	54	23	93
	4	w4	503	214	9	56	25	61
	5	w5	299	233	121	39	31	41
	6	w6	436	147	91	36	26	63
	7	w7	341	176	107	42	38	38
	8	w8	298	202	99	97	20	24
	9	w9	392	227	162	93	31	100
	10	w10	434	63	146	36	37	97
	11	w11	229	127	111	13	23	29
	12	w12	233	73	38	6	31	59
	13	w13	221	305	126	5	23	80
	14	w14	217	306	160	34	29	82
	15	w15	184	160	142	101	51	59
	16	w16	206	139	145	42	37	80
	17	w17	202	153	134	42	50	55
	18	w16	193	123	207	108	44	35
	19	w19	278	223	176	113	51	84
	20	w20	215	113	163	72	28	74
	21	w21	501	214	130	70	37	15
	22	w22	303	258	134	104	48	38
	23	w23	378	166	140	45	34	44
	24	w24	245	64	101	64	44	50
	25	w25	227	109	123	57	27	54
	26	w26	230	0	105	50	29	27
	27	w27	209	103	90	40	24	41
	28	w28	189	155	38	52	24	45
	29	w29	165	98	94	57	77	71
	30	w30	151	46	87	31	31	67
	31	w31	180	41	49	35	63	32
	32	w32	104	115	72	44	84	50
	33	w33	115	91	86	32	63	52
	34	w34	32	86	100	34	83	44
	35	w35	140	166	34	30	22	34
	36	w36	152	62	42	25	29	52
	37	w37	214	59	101	37	27	42
	38	w38	284	139	47	35	35	23

	39	w39	177	53	68	41	30	28
	40	w40	162	71	85	21	29	33
	41	w41	211	82	105	38	24	47
	42	w42	178	70	67	16	32	54
	43	w43	162	88	72	40	26	48
	44	w44	94	84	76	31	38	60
	45	w45	93	140	81	42	26	43
	46	w46	184	91	78	42	45	31
	47	w47	226	116	61	65	39	34
	48	w48	254	173	63	53	28	17
	49	w49	151	166	37	51	44	30
	50	w50	226	132	30	68	42	48
	51	w51	140	189	57	50	49	49
	52	w52	139	90	44	40	32	13
		Total	52137	21611	20499	12863	8415	11867

Matarara	Sembezeia	Nhambaml	Rupisse	IFLOMA	Rotanda	Moha	Total
0 a 4	0 a 4	0 a 4	0 a 4	0 a 4	0 a 4	0 a 4	0 a 4
24	41	12	56	23	16	27	836
59	63	42	50	31	22	24	1185
56	53	69	46	11	28	26	1254
48	47	11	34	27	26	20	1091
44	30	38	34	26	17	23	1005
19	36	10	27	21	20	24	767
22	58	6	25	24	17	26	861
40	56	11	37	21	23	23	811
39	80	61	46	26	18	26	1127
31	64	68	32	19	37	29	970
33	80	99	25	60	38	35	1111
35	71	110	17	52	25	22	1036
32	69	29	47	60	23	24	901
33	78	78	32	52	30	27	922
24	72	163	64	44	29	24	1004
39	66	53	51	53	33	47	961
70	37	54	83	53	28	47	927
41	62	102	50	60	26	44	1013
53	53	101	58	46	32	63	1092
49	24	88	51	35	15	42	909
32	32	68	43	29	12	36	814
17	39	37	24	24	6	32	790
32	23	33	62	30	6	24	822
44	35	54	32	33	14	25	789
25	47	30	33	32	7	29	645
21	40	21	30	14	8	21	568
16	18	23	22	18	15	36	573
20	20	38	22	10	7	31	528
15	10	25	21	11	5	32	451
15	21	19	9	7	4	23	442
25	40	17	24	6	2	12	440
12	40	19	13	4	2	16	381
17	22	15	18	9	3	16	434
11	28	28	15	18	5	13	402
12	26	16	23	5	3	14	354
32	17	29	16	9	7	15	367
20	13	19	21	8	8	25	421
15	19	31	30	16	12	27	438
15	13	38	21	12	3	30	375
13	24	21	16	15	3	24	442
31	28	37	18	17	7	26	565
15	28	15	15	10	7	36	614
29	20	22	35	23	7	32	662
35	41	48	40	24	13	37	696
15	33	30	45	30	13	33	648
27	31	52	34	22	8	34	700

24	23	34	30	21	7	32	606
25	30	0	25	33	7	32	556
28	52	0	21	21	16	42	589
19	37	5	37	33	18	42	808
33	52	81	17	38	13	45	817
25	27	25	29	34	14	41	809
23	46	37	37	49	29	54	702
34	55	84	65	54	32	97	1152
21	69	72	58	42	29	54	865
16	56	78	42	45	36	67	887
14	41	68	46	53	8	81	980
13	12	0	47	67	52	21	880
22	42	45	83	67	14	76	1159
21	76	88	22	76	10	36	1094
15	45	35	38	61	13	39	794
19	55	37	38	55	37	52	916
16	67	28	43	37	15	54	825
10	54	56	41	39	31	49	816
16	42	42	26	56	28	62	1032
14	46	40	38	37	17	63	804
49	66	43	67	64	24	52	1005
27	10	25	52	52	27	55	989
25	12	30	46	36	13	39	734
33	56	52	36	40	10	30	784
33	17	40	45	21	16	28	748
37	28	47	53	37	9	20	829
36	38	75	59	37	27	40	827
53	35	45	30	21	12	31	734
35	25	21	31	26	8	32	617
29	23	48	41	8	2	31	650
10	33	30	20	17	5	23	551
29	23	39	34	17	3	20	611
32	26	48	25	12	1	13	508
23	31	0	32	52	2	28	602
31	29	11	12	15	5	19	539
46	26	49	29	13	5	16	615
17	22	40	23	10	2	11	488
11	18	18	16	6	3	9	439
29	19	47	10	10	5	54	567
21	15	43	16	9	2	7	513
6	15	32	16	4	1	21	411
29	11	33	8	7	2	12	422
22	11	37	14	24	5	20	451
17	24	30	7	8	5	13	487
30	20	17	27	0	5	11	458
21	21	29	27	3	6	32	527
21	27	11	25	11	5	31	439
30	29	21	22	3	4	31	522
22	35	50	40	23	10	55	651
11	36	53	40	24	17	46	642

23	18	29	21	34	12	46	676
22	39	46	25	26	19	31	687
23	26	31	38	32	17	53	666
15	32	32	27	51	5	34	601
16	27	33	25	23	15	39	667
13	42	49	46	31	9	43	601
26	40	38	39	30	10	45	628
26	36	41	27	57	14	45	624
42	43	127	28	81	43	23	715
39	37	109	19	58	33	17	1222
61	33	92	23	35	34	23	1127
9	15	29	31	70	44	67	1072
15	53	132	24	34	33	11	1005
14	61	98	22	33	15	50	1009
26	85	70	10	41	36	50	933
32	75	12	23	55	58	78	1014
25	86	45	23	35	38	15	808
51	46	71	35	65	56	42	966
40	51	48	56	56	56	11	857
22	11	27	26	33	52	28	833
22	24	6	3	18	25	13	740
17	6	35	11	32	22	18	620
34	12	66	5	26	23	4	668
23	7	10	15	15	24	20	585
24	13	67	34	46	39	15	636
15	33	47	21	37	22	18	773
47	43	124	44	75	39	20	957
14	11	77	30	40	28	60	834
21	17	76	29	33	33	28	765
28	54	76	22	30	21	30	776
40	33	9	16	8	14	10	529
40	35	9	23	23	11	70	471
28	48	15	26	18	4	30	491
8	47	44	20	15	29	27	520
45	53	37	36	13	15	9	510
24	15	17	16	20	30	10	528
5	5	18	35	12	21	21	341
19	38	23	61	12	44	16	499
24	20	17	24	10	12	19	711
5	22	12	13	12	15	18	361
18	24	14	12	4	20	4	400
24	33	18	19	4	34	10	367
17	33	16	7	18	22	14	416
19	18	6	23	4	9	7	333
20	77	55	11	36	19	25	543
21	77	43	25	21	21	17	531
24	44	56	20	40	30	30	491
24	25	25	25	40	29	18	419
23	26	53	18	13	31	25	444
31	44	17	10	12	41	18	392

25	28	70	20	22	25	30	460
15	19	37	34	29	30	25	407
9	43	49	18	17	9	20	384
12	38	25	6	13	22	13	347
22	24	10	16	12	36	11	383
17	24	19	15	23	44	14	435
17	30	41	21	23	24	47	699
24	33	16	25	30	27	60	793
16	34	60	23	19	20	50	706
18	37	88	28	28	33	22	887
26	0	81	39	56	31	60	457
39	57	158	43	27	42	40	1134
0	73	2	40	0	0	40	878
15	51	12	28	12	0	40	692
31	53	101	33	35	0	40	830
0	57	0	18	37	0	50	683
0	52	81	47	39	0	40	733
0	68	14	53	0	0	40	606
31	64	78	44	17	0	40	759
0	68	0	41	16	0	0	731
52	0	0	25	0	0	31	538
2	0	0	34	22	0	0	535
32	0	113	19	13	28	28	699
51	0	0	36	18	0	0	471
54	0	0	32	0	22	30	629
58	47	0	25	0	18	0	809
1	43	52	25	16	21	0	752
38	23	48	40	12	19	21	763
63	35	75	25	0	22	19	803
38	23	0	23	0	19	0	567
0	21	0	13	51	0	20	646
29	18	0	14	10	0	22	516
0	16	0	25	0	0	23	392
0	25	0	28	9	26	27	523
44	7	0	13	0	0	0	390
25	12	0	11	4	0	17	335
0	0	0	13	2	31	0	310
29	0	0	12	0	0	11	347
47	0	0	10	0	22	16	385
25	0	0	11	0	0	19	255
0	8	0	0	0	0	0	82
0	0	0	0	0	0	0	30
0	0	0	4	0	0	6	118
0	0	0	15	0	0	0	204
0	0	0	0	0	0	0	63
0	0	20	15	3	0	8	420
0	0	21	0	1	0	10	419
0	0	37	0	4	0	11	406
0	0	17	0	3	14	19	323
33	11	33	18	0	9	13	435

40	11	26	20	1	17	14	406
29	18	31	18	3	11	0	432
21	0	2	17	0	16	0	286
45	26	31	23	5	11	19	483
42	23	48	23	8	15	26	556
42	23	30	18	21	11	42	651
27	41	37	12	9	22	30	544
22	42	64	17	9	21	17	725
41	46	120	17	13	29	60	842
40	49	58	15	8	1	27	800
39	43	98	38	12	23	11	574
29	29	31	21	8	26	48	645
24	41	104	56	19	20	34	257
59	42	159	50	17	22	32	1158
56	78	69	46	14	28	26	1245
48	78	11	34	17	31	19	1106
44	62	132	34	23	17	28	1104
19	36	10	27	27	20	19	957
22	58	74	25	21	17	26	984
40	61	12	37	27	26	25	968
39	91	13	46	26	18	25	1263
31	122	23	32	28	37	21	1107
33	46	99	31	28	38	31	838
35	10	81	17	26	25	22	656
32	69	29	47	60	23	28	1048
33	80	60	32	52	12	31	1128
24	72	163	64	44	29	21	1115
39	1	93	51	53	17	32	935
70	44	54	83	53	13	47	1000
41	43	102	50	60	26	44	1076
53	56	31	58	46	10	63	1242
49	59	132	39	35	22	42	1043
32	63	0	43	29	18	36	1188
17	42	79	24	24	12	32	1115
32	54	56	62	30	13	24	1078
44	52	0	32	33	12	25	766
25	41	54	33	32	16	29	827
21	31	54	30	14	5	30	626
16	48	23	22	18	12	36	682
20	20	33	22	10	7	31	646
15	10	25	21	21	6	26	686
37	21	11	9	7	2	23	523
25	40	17	24	6	2	12	526
12	40	19	13	3	2	16	574
17	15	21	18	2	1	20	533
11	28	28	14	18	2	21	501
12	26	16	23	5	3	14	525
32	17	36	16	9	7	15	494
20	11	19	21	8	1	25	585
15	19	18	30	16	14	27	701

15	20	15	21	12	3	30	513
13	13	25	16	15	2	24	509
56	24	40	18	17	8	26	696
56	32	32	15	10	5	36	603
29	33	32	35	23	7	32	627
26	51	48	24	24	6	37	599
15	33	30	45	28	13	33	622
18	31	52	34	34	8	25	673
22	23	34	30	33	10	31	725
27	30	0	25	31	7	32	740
33	52	0	21	21	16	17	640
35	37	5	37	33	18	42	753
24	52	81	32	38	12	45	818
35	27	25	33	34	14	41	566
6657	8937	10226	7312	6117	4072	7244	177957
